# Novel real-time optical imaging modalities for the detection of neoplastic lesions in urology: a systematic review

**DOI:** 10.1007/s00464-018-6578-1

**Published:** 2018-11-12

**Authors:** Oliver Brunckhorst, Qi Jia Ong, Daniel Elson, Erik Mayer

**Affiliations:** 1 Department of Surgery and Cancer, Imperial College London, St Mary’ Hospital Campus, 10th Floor QEQM Building, Praed Street, London, W2 1NY UK; 2 Hamlyn Centre for Robotic Surgery, Institute of Global Health Innovation, Imperial College London, London, UK

**Keywords:** Optical imaging, Diagnostic imaging, Neoplasm, Urological malignancy

## Abstract

**Background:**

Current optical diagnostic techniques for malignancies are limited in their diagnostic accuracy and lack the ability to further characterise disease, leading to the rapidly increasing development of novel imaging methods within urology. This systematic review critically appraises the literature for novel imagining modalities, in the detection and staging of urological cancer and assesses their effectiveness via their utility and accuracy.

**Methods:**

A systematic literature search utilising MEDLINE, EMBASE and Cochrane Library Database was conducted from 1970 to September 2018 by two independent reviewers. Studies were included if they assessed real-time imaging modalities not already approved in guidelines, in vivo and in humans. Outcome measures included diagnostic accuracy and utility parameters, including feasibility and cost.

**Results:**

Of 5475 articles identified from screening, a final 46 were included. Imaging modalities for bladder cancer included optical coherence tomography (OCT), confocal laser endomicroscopy, autofluorescence and spectroscopic techniques. OCT was the most widely investigated, with 12 studies demonstrating improvements in overall diagnostic accuracy (sensitivity 74.5–100% and specificity 60–98.5%). Upper urinary tract malignancy diagnosis was assessed using photodynamic diagnosis (PDD), narrow band imaging, optical coherence tomography and confocal laser endomicroscopy. Only PDD demonstrated consistent improvements in overall diagnostic accuracy in five trials (sensitivity 94–96% and specificity 96.6–100%). Limited evidence for optical coherence tomography in percutaneous renal biopsy was identified, with anecdotal evidence for any modality in penile cancer.

**Conclusions:**

Evidence supporting the efficacy for identified novel imaging modalities remains limited at present. However, OCT for bladder cancer and PDD in upper tract malignancy demonstrate the best potential for improvement in overall diagnostic accuracy. OCT may additionally aid intraoperative decision making via real-time staging of disease. Both modalities require ongoing investigation through larger, well-conducted clinical trials to assess their diagnostic accuracy, use as an intraoperative staging aid and how to best utilise them within clinical practice.

Advances in established imaging technologies such as computed tomography, positron emission tomography and magnetic resonance imaging are providing increasingly accurate and reliable information for the detection and staging of all types of cancers [1]. However, real-time optical imaging modalities involving endoscopic or minimally invasive techniques in various cancers lack the ability to provide this level of information and offer varying diagnostic accuracies [2, 3]. This is important as false-negative results put patients at risk of undetected cancer and progression, whilst false-positive results lead to unnecessary biopsies, resulting in stress to the patient with a burden of unnecessary care [4]. These issues are pertinent in urological malignancies where current standards of practice such as white light cystoscopy are user dependent, with varying sensitivities and specificities [5, 6]. Furthermore, visual appearance of bladder lesions is known to be unreliable for further characterisation of lesions with regard to their grade and/or level of invasion which can impact treatment decisions [7]. Therefore, a need for additional real-time optic imaging modalities in urology exists, to improve both diagnostic accuracy and characterisation of tumours identified.

Novel optical imaging modalities currently being developed and assessed may provide this much-needed addition to support real-time diagnostic imaging. These utilise visible, ultraviolet or infrared light emitted from a light source such as xenon or laser to assess anatomic or chemical properties of tissues, with or without the use of endogenous or exogenous fluorophores [8]. Advances in technology and increasing interest surrounding these modalities have meant a shift of focus from laboratory-based to clinical applicability research [9]. Applicable to cystoscopy some modalities such as photodynamic diagnosis (PDD) and narrow band imaging (NBI) have already demonstrated improved diagnostic accuracies and have, therefore, established themselves within urological guidelines [10–13]. However, the evidence is less clear in other more novel optical imaging modalities and in other urological malignancies where the technology is only now allowing for increasing clinical assessment. This systematic review therefore aims to,

1. provide a critical overview of the current literature with regards to the use of novel optical imagining modalities, used for the detection and staging of cancer in urology. Novel, for the context of this study, being defined as those not approved in current urological guidelines.
2. assess the effectiveness of identified modalities through their feasibility, diagnostic accuracy, cost and utility
3. identify future areas of research based on the current literature

## Materials and methods

This systematic review was performed following guidelines defined in the Preferred Reporting Items for Systematic Reviews and Meta-Analyses (PRISMA) statement [14] and prospectively registered, PROSPERO Registration No.: CRD42017084172.

### Study eligibility criteria

Original research studies describing the use of novel imaging techniques with applicability in detection or staging/grading of urological cancer or pre-malignant disease processes were included in this study. Only studies describing use of imaging systems used intraoperatively in real-time were included. Novel imaging for the context of this study was defined as imaging modalities not described in international or United Kingdom urological guidelines for the detection of cancer including European Association of Urology (EAU), American Urological Association (AUA) and National Institute for Health and Care Excellence (NICE) guidelines. Only in vivo, human subject studies were included with no limitation on study type including all experimental and observational study types. Exclusion criteria were ex vivo studies, in vitro studies, animal studies, comments, reviews articles, letters, non-English articles and paediatric studies. Additionally, studies describing the use of imaging modalities as a guidance for the treatment and excision of confirmed cancers, as opposed to guiding intraoperative diagnosis, were excluded from the review.

### Information sources and search

A comprehensive search was performed from January 1970 to 28th September 2018. MEDLINE (via Pubmed), EMBASE and the Cochrane Library Database were initially searched utilising broad MeSH terms including ‘Optical Imaging’ and ‘Diagnostic Imaging’ combined with urology key terms; ‘urology OR urological OR urinary OR bladder OR renal OR kidney OR ureter OR ureteric OR upper urinary tract’. Once imaging modalities were identified, each was searched against key urology terms. Subsequently, a reference review of identified articles and reviews was conducted to identify any pertinent articles. Grey literature was searched via guidelines from EAU, AUA and NICE and ongoing clinical trials through ClinicalTrials.gov, The ISRCTN registry and the World Health Organisation International Clinical Trials Registry Platform portal. Authors of trials were contacted for preliminary or unpublished results for inclusion in the review. Full search strategy and results are provided in Appendix A.

### Study selection

Two reviewers (OB and QO) independently identified potentially relevant articles that arose from the search strategy once duplicates were removed. An initial title and abstract screening was conducted with full text of each potentially relevant article subsequently assessed against the inclusion criteria. All discrepancies were discussed until 100% agreement was achieved.

### Data collection and data items

Data extraction was independently conducted by two reviewers (OB and QO) onto a pre-defined extraction sheet. Certain data were extracted from all studies including study type, number of participants, participant demographics including tumour stage/grade, novel imaging system utilised, and procedure type assessed. Primary outcome measures extracted for assessment of the effectiveness of a diagnostic modality included quantitative measures of accuracy via sensitivity, specificity, positive predictive value (PPV), and negative predictive value (NPV). Secondary outcome measures were quantitative and qualitative data on utility including feasibility, cost, stage of development and use with standard operative equipment.

### Risk of bias assessment

Individual studies were assessed for risk of bias utilising the QUADAS-2 tool [15]. Initial piloting led to removal of one signalling question regarding pre-defined test threshold for the index test, as this was not applicable. The final tool was used on all studies with subsequent summary graph production via RevMan 5.3 software. The GRADE tool was utilised subsequently for overall assessment of study quality for recommendation of use [16].

## Results

### Study selection

**Fig. 1:**
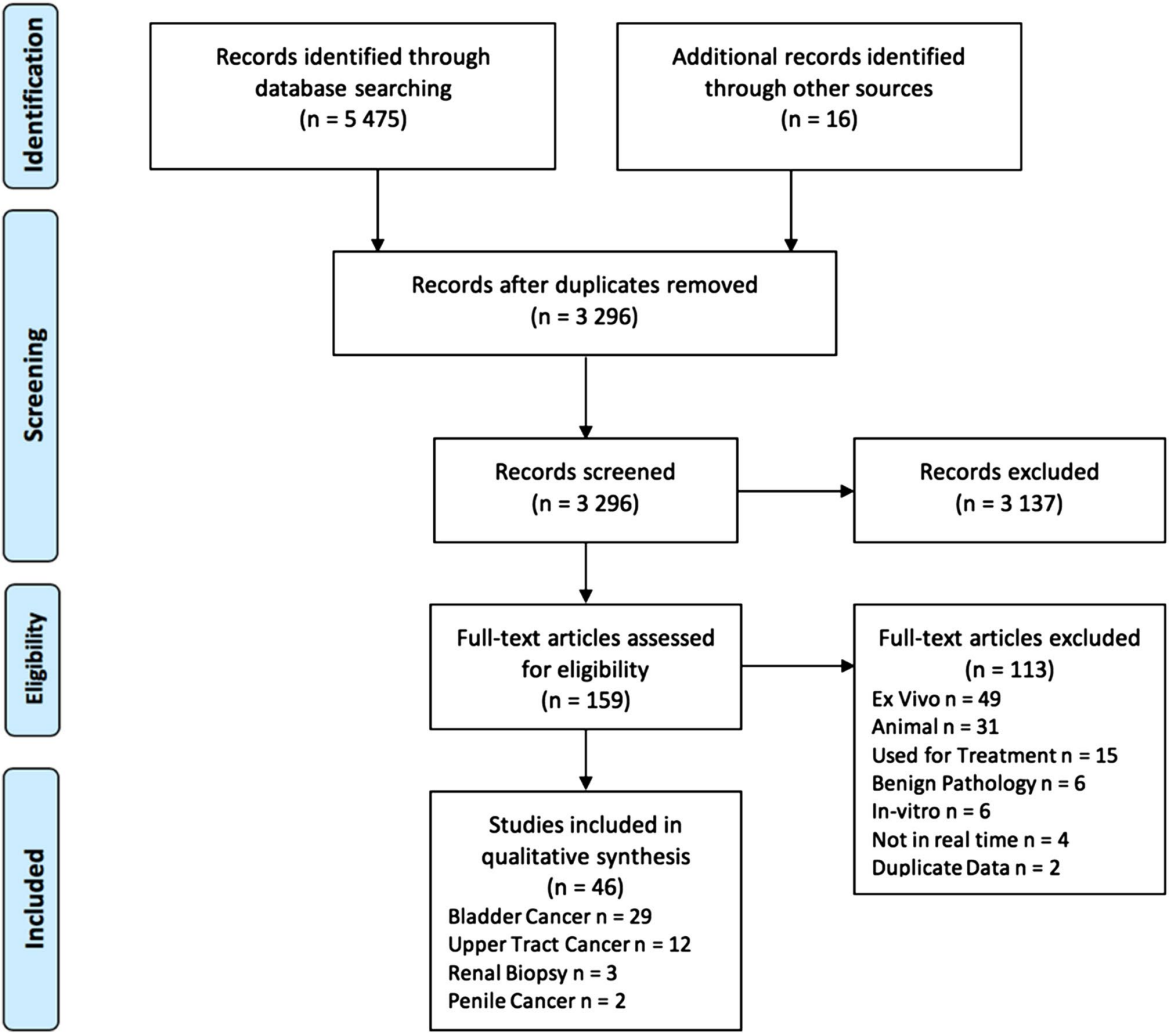
PRISMA diagram for study selection

A total of 5475 articles were identified through the literature search, with 16 articles identified via reference review. Duplicate removal and initial screening excluded 4928 articles. Of the 148 full text articles assessed for eligibility, 46 articles were included in the review (Fig. 1).

### Study characteristics and result synthesis

Selected articles consisted of experimental studies assessing the utility and diagnostic accuracy of novel imaging modalities in urological cancers. Results were classified into bladder, upper urinary tract, renal and penile cancer and further subdivided by imaging modality.

### Bladder cancer

#### Optical coherence tomography

With twelve studies assessing its use in bladder cancer (Table 1), optical coherence tomography (OCT) was the most widely studied imaging modality with a total of 566 patients investigated [17–28]. OCT utilises near-infrared light to measure the unique backscattering properties of different tissue layers of the bladder wall providing a real-time cross-sectional image with resolutions of 10–20 µm and depth of penetration of 1–2 mm [4]. Majority of studies produced a lateral scanning technique to produce a two-dimensional B-scan (analogous to ultrasound) introducing some control requirements of the probe. With regards to utility, all studies confirmed the feasibility of utilising OCT in vivo for real-time diagnosis of bladder malignancy; however, studies varied widely in the equipment utilised, with central wavelengths in the range of 830–1310 nm. The majority of equipment had an acquisition time of 1.5 s (1–3 s) with an image output of 200 × 200 pixels. Studies utilised different OCT probes; however, all were 2.7 mm and utilised with standard cystoscopy equipment, requiring only an additional computer system. Most utilised locally developed OCT systems, with only four studies using a commercially available system (Niris Imaging System), affecting the widespread uptake of this imaging modality [19, 20, 24, 25]. No studies discussed any cost-analysis for OCT.

**Table 1:** Summary of novel imaging modalities for bladder cancer

Imaging modality	Index test	Ref. test	No. of patients	Patient’s histology	Imaging modality technical details	Sensitivity (%)		Specificity (%)		PPV (%)		NPV (%)		Cost	Utility
						Ind	Ref	Ind	Ref	Ind	Ref	Ind	Ref		
OCT															
Gladkova et al. (2011) [17]	OCT	CP OCT	116	TCC—16 Tis—10 Ta-T1-6 Ben—100	Wavelength 1300 nm, resolution 15 µm, acquisition time 2 s, image format 200 × 200 pixels	81.2	93.7	70	84	30.2	48.3	95.1	98	×	Prototype system
Gladkova et al. (2013) [18]	OCT	CP OCT	26	TCC (Tis-T2a)—26	Wavelength 1300 nm, resolution 15 µm, acquisition time 2 s, image format not discussed	74.5	89.7	70.8	91.6	58.3	85.4	83.5	94.2	×	Prototype system
Goh et al. (2008) [19]	OCT	–	32	TCC (Ta-T2)—30 Ben—2	Niris imaging system, 1310 nm, resolution 10–20 µm, acquisition time 1.5 s, image format 200 × 200 pixels	93,5	–	88	–	85.2	–	95.2	–	×	Comm. available
Karl et al. (2010) [20]	OCT	–	52	Not specified	Wavelength 1300 nm, resolution 10–20 µm, acquisition time 1.5 s, image format 200 × 200 pixels	100	–	65	–	31.1	–	100	–	×	Comm. available
Kiselva et al. (2015) [21]	OCT	–	73	TCC (Tis-T2a)—32 Ben—41	Wavelength 1315 nm, resolution 15 µm, acquisition time 3 s, image format 200 × 256 pixels	86	–	68	–	–	–	–	–	×	Prototype system
Manyak et al. (2005) [22]	OCT	–	24	Not specified	Wavelength 980, acquisition time 1.5 s, image format 200 × 200 pixels, resolution not discussed	100	–	89	–	75	–	59	–	×	Prototype system
Ren et al. (2009) [23]	OCT	WLC	56	TCC—39 Tis—3 < T2-21 ≥ T2-15 Ben—17	Wavelength 1320 nm, no resolution, acquisition time or image format discussed	96.2	69.8	89.5	73.7	89.4	71.2	96.2	72.4	×	Prototype system
Schmidbauer et al. (2009) [24]	PDD + OCT	WLC	66	TCC (Tis-T2)—58 Ben—8	Niris imaging system, 1310 nm, resolution 15 µm, acquisition time 1.5 s, image format 200 × 200 pixels	97.5	69.3	97.9	83.7	96.4	77.9	97.9	76.7	×	Comm. available
Sengottayan et al. (2008) [25]	OCT	–	32	TCC (Ta-T2)—32	Niris imaging system, 1310 nm, resolution 10–20 µm, acquisition time 1.5 s, image format not discussed	–	–	–	–	89	–	100	–	×	Comm. available
Sergeev et al. (1997) [26]	OCT	–	3	TCC—3	Wavelength 830 nm, resolution 20 µm, acquisition time 1 s, image format 200 × 200 pixel	–	–	–	–	–	–	–	–	×	Prototype system
Wang et al. (2007) [27]	OCT	–	20	Not specified	Wavelength 1320 nm, resolution 10 µm, acquision time and image not discussed	91	–	80	–	–	–	–	–	×	Prototype system
Zagaynova et al. (2002) [28]	OCT	-	66	TCC (T1-T3)—20 SCC (T2-3)—8 Adenocarcinoma—2 Ben—36	Wavelength 1270 nm, resolution 10–20 µm, acquisition time 1.5 s, image format 200 × 200 pixels	–	–	–	–	–	–	–	–	×	Prototype system
CLE															
Adams et al. (2011) [29]	CLE	–	67	TCC—52 Low grade—22 High grade—22 Tis—8 Ben—17	Cellvizio system, 488 nm laser, resolution 1 µm, slice thick 10 µm	–	–	–	–	–	–	–	–	×	Comm. available
Liem et al. (2018) [32]	CLE	WLC	53	Patient specific data not Specified	Celvizio system, 488 nm laser, resolution 1 µm	76%	54%	76%	71%	–	–	–	–	×	Comm. available
Naya et al. (2018) [33]	CLE	WLC	1	TCC (Tis)—1	Celvizio system, no further technical details provided	–	–	–	–	–	–	–	–	×	Comm. available
Sonn et al. (2009) [30]	CLE	–	27	TCC—19 Low grade—9 High grade—9 Tis—1 Ben—8	Cellvizio system, 488 nm laser, resolution 1 µm, slice thick 10 µm	–	–	–	–	–	–	–	–	×	Comm. available
Wu et al. (2011) [31]	CLE	–	66	Not specified	Cellvizio system, 488 nm laser resolution 1 µm, slice thick 10 µm	–	–	–	–	–	–	–	–	×	Comm. available
Autofluorescence															
Anidjar et al. (1996) [34]	AF	–	25	TCC (Tis-T3)—25	Three lasers used, 480 nm, 337 nm and 308 nm, recording AF 320–600 nm	–	–	–	–	–	–	–	–	×	Prototype system
Jacobson et al. (2012) [35]	AF	–	21	TCC (Ta-T2)—21	Near infrared monochromatic 650 nm laser	–	–	–	–	–	–	–	–	×	Prototype system
Koenig et al. (1996) [36]	AF	–	53	Not specified	Nitrogen 337 nm laser, recording spectrum 300–800 nm	97	–	98	–	93	–	99	–	×	Prototype system
Koenig (1998) [37]	AF	–	75	Not specified	Nitrogen laser 385 nm and 455 nm, recording spectrum 300–800 nm	–	–	–	–	–	–	–	–	×	Prototype system
Kriegmair et al. (2017) [38]	AF	WLC	25	Not specified	Wavelength 440 nm, recording spectrum 480–780 nm	96.7	86.7	53.9	69.2	70.7	76.5	93.3	81.8	×	Comm. available
Schafauer et al. (2013) [39]	AF	–	14	TCC—7 Tis—2 Ta—4 T2—1 Ben—7	Excimer laser 308 nm, recording spectrum 300–600 nm	100	–	–	–	–	–	–	–	×	Prototype system
Szygula et al. (2004) [40]	AF	PDD	229	TCC (all T1)—92 Ben—137	Blue laser light irradiation via Xillix LIFE diagnostic system	97.8	90.9	70.1	66.6	–	–	–	–	×	Comm. available
Spectroscopies															
Koening et al. (1998) [41]	DRS	–	14	TCC (Tis-T2)—14	Light probe emitting 400–700 nm with 0.6 mm optical recording probe	91	–	60	–	63	–	90	–	×	Prototype system
Mourant et al. (1995) [42]	DRS	–	10	Not specified	Light probe emitting 250–1000 nm with 0.2 mm optical recording probe	100	–	97	–	–	–	–	–	×	Prototype system
Draga et al. (2010) [43]	Raman	PDD	38	TCC (Ta-T2)—38	785 nm diode laser, collecting spectra between 400 and 1800 nm	85	85	79	69	85.3	–	65.7	–	×	Prototype system
Endocystoscopy															
Lovisa et al. (2010) [44]	HMC	–	78	Not specified	Rigid cystoscopy with magnification power between 30 and 650-fold	97	–	85	–	91.4	–	94.4	–	×	Prototype system
Ohigashi et al. (2010) [45]	HMC	–	5	TCC (Ta-T1)—5	3.2 mm probe through cystoscope with 450-fold magnification	–	–	–	–	–	–	–	–	×	Prototype system

Index—*AF* Autofluorescence, *Ben* Benign, *CLE* confocal laser endomicroscopy, *CP OCT* cross polarization optical coherence tomography, *DRS* diffuse reflectance spectroscopy, *HMC* high magnification cystoscopy, *OCT* optical coherence tomography, *PDD* photodynamic diagnosis, *SCC* squamous cell carcinoma, *TCC* transitional cell carcinoma, *WLC* white light cystoscopy

**Fig. 2:**
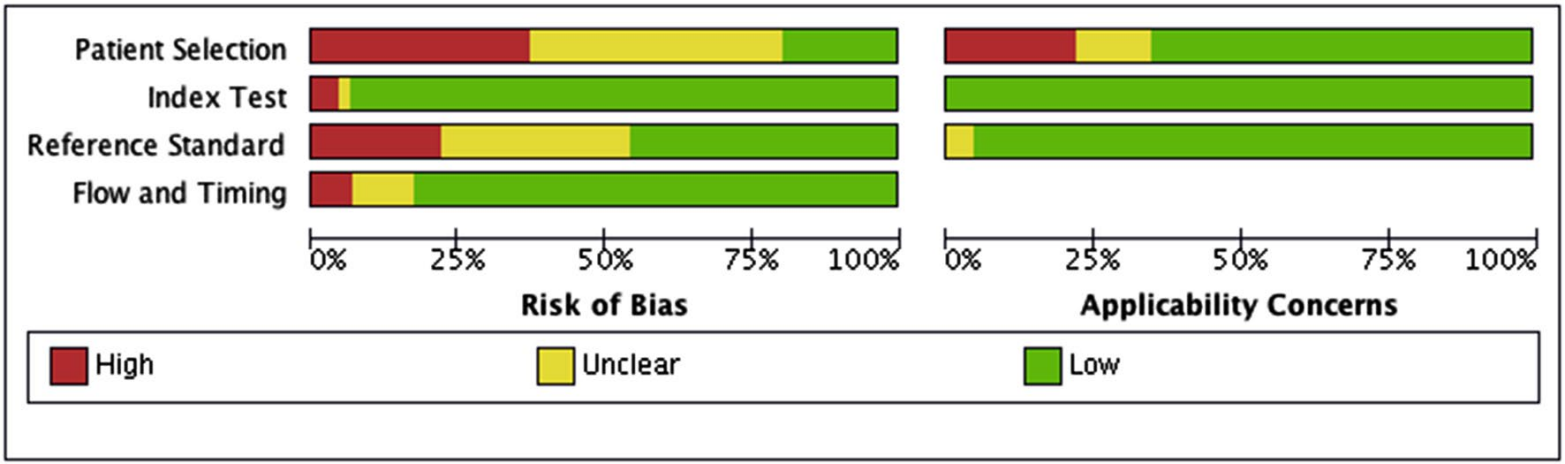
QADAS-2 risk of bias assessment summary table

Diagnostic accuracy of OCT was assessed by ten studies [17–25, 27]. These studies assessed transitional cell bladder carcinoma (TCC), ranging from non-muscle invasive bladder cancer (Tis, Ta and T1 disease) to T2 disease. Sensitivity and specificity for the use of OCT after white light cystoscopy for differentiation between benign and malignant lesions varied from 74.5 to 100% and 60 to 98.5%, respectively. The PPV and NPV varied between 30.2–89.4% and 72.4–100%. A single study of 66 patients assessed the use of OCT combined with blue light cystoscopy with a sensitivity and specificity of 97.5% and 97.9% and PPV and NPV of 96.4% and 97.9%, an improvement on white light or blue light cystoscopy alone [24]. Only three studies assessed the diagnostic accuracy of OCT for staging of disease with sensitivity and specificity of 88.9–90% and 89% for carcinoma in situ (CIS) and 75–100% and 89–97% for muscle invasion (T1-2 disease) (19, 23, 25). Risk of bias assessment (Fig. 2) revealed consistent patient selection bias, with the use of consecutive patients not included or specified in ten studies.

#### Confocal laser endomicroscopy

Confocal laser endomicroscopy (CLE) utilises a fibre-optic imaging probe in contact with tissue and a laser-excited fluorescent contrast agent such as fluorescein to provide real-time depth-sectioned microscopic imaging close to the tissue surface [9]. Its high resolution of 1–5 µm provides en-face imaging to the cellular level which could be used for tissue grading; however, lacks the tissue penetrance (40–70 µm) to accurately assess depth of invasion. Five studies with 214 patients combined utilised CLE in vivo and in real-time [29–33]. The majority of studies were largely human feasibility studies to demonstrate differences between malignant and non-malignant non-muscle invasive bladder cancer (NMIBC). Additionally, differentiation between low- and high-grade tumours was sought in these studies. Only a single study of 53 patients has assessed diagnostic accuracy, specifically for grading of identified lesions identifying a sensitivity of 76% and 70% for low- and high-grade lesions, respectively [32]. Overall diagnostic specificity was identified at 96% for the cohort. All studies identified utilised a commercially available imaging system (Cellvizio® system) within their study protocol, with either 2.6 mm or 1.4 mm probes compatible with standard cystoscopy equipment. Consistent patient selection bias was identified due to lack of consecutive patient recruitment in three of the studies (Appendix B).

#### Autofluorescence

Autofluorescence relies on the intrinsic fluorescence of tissues resulting from naturally occurring fluorophores such as elastin, collagen, NADH, FAD etc. when excited by ultraviolet, visible or near-infrared light [8] as opposed to the use of an extrinsic fluorophore. Seven studies with 494 participants meeting the inclusion criteria were included [34–40]. Studies varied with respect to the excitation source from nitrogen to excimer laser, with wavelengths from 308 to 650 nm, making direct comparison between studies difficult. However, all were feasibly utilised with three being conducted entirely with commercially available equipment. Diagnostic accuracy was assessed in four studies, assessing both NMIBC and T2-3 disease on histopathologically demonstrated TCC. Sensitivities and specificities for differentiation between benign and malignant varied from 96.7 to 100% and 53.9–98%, respectively. PPV and NPV were found to be between 70.7–93% and 93.3–99%. No assessments between staging/grading of cancers or costs were performed. Consistent unclear or high patient selection bias due to consecutive patient enrolment was seen in all seven studies on risk of bias assessment.

#### Diffuse reflectance spectroscopy

Diffuse reflectance spectroscopy utilises a light source and a detection fibre in contact with the tissue to pick up differences in light backscattered from beneath the surface of the tissue [41]. Two small studies have utilised this in vivo for both NMIBC and T2 disease, with a total of only 24 patients [41, 42]. These were mostly proof of concept studies confirming feasibility and utilising prototype equipment only. However, both analysed diagnostic accuracy, identifying sensitivity and specificity of 91–100% and 60–97%. No discussion regarding cost was made with no consistent trends on risk of bias assessment.

#### Raman spectroscopy

Raman spectroscopy aims to give a molecular fingerprint via a Raman probe which detects Raman scattered light, shifted to longer wavelengths through interaction with molecular vibrational energy levels, giving a spectrum of peaks characteristic to a tissue type [43]. Only a single study of 38 participants with Ta-T2 disease, has been conducted in vivo, relying on a prototype system [43]. This was predominantly conducted to identify reliable peaks for bladder cancer and to confirm feasibility. The study gives a sensitivity of 85% and specificity of 79% for benign versus malignant tissue and did not discuss cost of equipment. Acquisition times for signals were long and collected at between 1 and 5 s, with no difference in diagnostic accuracy between shorter and longer times. This single study possessed selection and flow bias with not all patients included in the analysis.

#### High magnification cystoscopy

Endocystoscopy gives high magnification views through a standard cystoscope of up to 650-fold magnifying power, providing a more detailed cellular and vascular image of the tissue. Two studies utilised prototype cystoscopes to assess this in vivo during PDD and white light cystoscopy [44, 45]. A single study of 78 patients assessed diagnostic accuracy in urothelial dysplasia and TCC patients with NMIBC and T2 disease. This identified an overall sensitivity between benign and malignant of 97% and specificity of 85% for lesions already identified through blue light [44]. No discussions regarding cost were made in either study with no trends on risk of bias seen.

### Upper urinary tract malignancy

#### Optical coherence tomography

Upper tract urothelial carcinoma (UTUC) diagnosis is limited by poor accuracy of standard ureteroscopy and inconclusive histology samples leading to several optical imaging modalities being recently investigated (Table 2). OCT has been assessed for its use in ureteroscopy in two small studies with 34 patients combined, including both non-invasive (Ta and Tis) and invasive (T1-4) UTUC in their evaluation [46, 47]. Both utilised commercially available OCT systems (C7-XR OCT system) which provide an automatic 360-degree image of a longitudinal trajectory, when used with standard ureteroscopy equipment. The larger study of 26 patients assessed diagnostic accuracy, specifically to staging of disease, with a sensitivity and specificity of 91.7% and 78.6% and a PPV and NPV of 92% and 100%. However, no discussion regarding cost was made in either. Risk of bias assessment demonstrates reference standard bias due to histopathology being a known poor gold-standard in upper tract malignancy.

**Table 2:** Summary of novel imaging modalities for upper urinary tract malignancy

Imaging modality	Index test	Ref. test	No. of patients	Patient’s histology	Imaging modality technical details	Sensitivity (%)		Specificity (%)		PPV (%)		NPV (%)		Cost	Utility
						Ind	Ref	Ind	Ref	Ind	Ref	Ind	Ref		
OCT															
Bus et al. (2013) [46]	OCT	–	8	UTUC—8 Tis-Ta—4 T1-3-4	C7-XR OCT system, 1300 nm longitudinal 54 mm and 360-degree trajectory taking 5.4 s	–	–	–	–	–	–	–	–	×	Comm. available
Bus et al. (2016) [47]	OCT	–	26	UTUC—24 Tis-Ta—14 T1-T4-12	C7-XR OCT system, 1300 nm longitudinal 54 mm and 360-degree trajectory taking 5.4 s	91.7	–	78.6	–	92	–	100	–	×	Comm. available
CLE															
Breda et al. (2017) [50]	CLE	–	14	UTUC—12 Low grade—6 High grade—5 Tis—1 Unknown—2	Celvizio system, no further technical details provided	–	–	–	–	–	–	–	–	×	Comm. available
Bui et al. (2015) [48]	CLE	–	14	UTUC—7 Low grade—4 High grade—3 Ben—7	0.85 mm probe, resolution 3.5 µm, field of view 320 µm, depth 50 µm	–	–	–	–	–	–	–	–	×	Prototype system
Villa et al. (2016) [49]	CLE	–	11	UTUC—10 Low Grade—7 High Grade—3 Ben—1	Cellvizio system, 488 nm laser resolution 3.5 µm, depth 40–70 µm	–	–	–	–	–	–	–	–	×	Comm. available
PDD															
Aboumarzouk et al. (2012) [52]	PDD	WLU	32	UTUC—25 Tis-Ta—23 T1-2 Ben—7	Xenon blue light 380–440 nm, oral 5-ALA pre-operatively	96	80	100	86	100	95	88	55	×	Comm. available
Aboumarzouk et al. (2013) [53]	PDD	WLU	30	UTUC—17 Tis-Ta—16 T2-1 Ben—13	Xenon blue light 380–440 nm, oral 5-ALA pre-operatively	94	82.4	100	100	100	100	93	81	×	Comm. available
Ahmad et al. (2012) [54]	PDD	-	26	Not specified	Xenon blue light 380–440 nm, oral 5-ALA pre-operatively	-	-	-	-	-	-	-	-	×	Comm. available
Kata et al. (2016) [55]	PDD	WLU	54	Not specified	Xenon blue light 380–440 nm, oral 5-ALA pre-operatively	95.8	53.5	96.6	95.2	95.8	88.5	96.6	75	✓	Comm. available
Somani et al. (2010) [56]	PDD	-	4	UTUC (Ta)—4	Xenon blue light 380–440 nm, oral 5-ALA pre-operatively	-	-	-	-	-	-	-	-	×	Comm. available
NBI															
Chan et al. (2014) [57]	NBI	WLU	7	UTUC—7 Ta—2 T1-3-5	Olympus NBI system, 415 nm blue light and 540 nm green light	-	-	-	-	-	-	-	-	×	Comm. available
Traxer et al. (2011) [58]	NBI	WLU	27	UTUC (Ta)—20 Ben—1 Invalid Biopsy—6	Olympus NBI system, 415 nm blue light and 540 nm green light	-	-	-	-	-	-	-	-	×	Comm. available

Index—*Ben* benign, *CLE* confocal laser endomicroscopy, *OCT* optical coherence tomography, *NBI* narrow band imaging, *PDD* photodynamic diagnosis, *UTUC* upper tract urothelial carcinoma, *WLU* white light ureteroscopy, 5-*ALA* 5-aminolevulinic acid

#### Confocal laser endomicroscopy

CLE was assessed in three studies meeting inclusion criteria [48–50]. Studies had a combined 39 UTUC patients and were largely conducted to assess feasibility of differentiation between low- and high-grade tumours. IV fluorescein prior to the procedure was used in both, with two utilising a commercially available system (Cellvizio® system). Two studies claim that CLE was able to differentiate between benign/malignant and low/high-grade tissue without further no quantitative data for diagnostic accuracy. One study assessed correspondence between CLE images and biopsy results, identifying this as 100% for low-grade lesions, 83% for high-grade and 100% for in situ disease in a limited cohort of 14 patients [50]. However, no formal sensitivity or specificity was provided with no study discussing cost. Unclear significance of patient selection bias was revealed in all studies with concerns regarding histopathology as the reference standard.

#### Photodynamic diagnosis

Whilst well established in bladder cancer, PDD is not yet recommended in urological guidelines for upper tract malignancy [51]. PDD requires administration of a preoperative fluorophore which fluoreses when exposed to blue light (380–480 nm) intraoperatively. Five retrospective and prospective studies were identified which assessed PDD in UTUC with a total of 146 patients [52–56]. Patients stage at assessment ranged from Tis and Ta to T2 disease. All studies used oral 5-aminolevulinic acid along with commercially available equipment commonly used for bladder PDD (Xenon blue light, 380–440 nm). Three studies assessed diagnostic accuracy, with sensitivity and specificity in the range of 94–96% and 96.6–100%. PPV and NPV were seen at 95.8–100% and 88–96.6%. All demonstrated improved accuracy when compared to white light ureteroscopy. One study discussed the cost of PDD with a per patient price of £110 for the fluorophore and a one-off cost of £12,000 for the stack system. However, no study assessed cost effectiveness or total cost per-procedure. All five studies discussed above were conducted at a single centre with three studies demonstrating concerns regarding patient selection bias with all having reference standard concerns.

#### Narrow band imaging

Similarly to PDD, whilst narrow band imaging (NBI) is recommended for bladder malignancy, this is not the case for upper tract disease [51]. NBI filters out red light from the white light spectrum, as well as filtering the remining light into narrow blue and green bands at 415 nm and 540 nm which enhances mucosal and submucosal vasculature [4]. Within upper tract malignancy, only two studies were identified that matched inclusion criteria [57, 58]. These studies were small with a combined 35 patients with UTUC (Ta-T3 disease). They were largely feasibility studies utilising commercially available equipment, however, made no analysis on diagnostic accuracy or cost of NBI in upper tract disease. Both studies demonstrated unclear or high risk of bias on patient selection and reference standard bias.

### Renal cancer

#### Optical coherence tomography

OCT has recently been investigated in renal cell cancer (RCC) diagnosis for percutaneous biopsies of solid masses. An OCT probe is introduced via the puncture trocar with images of the tumour obtained to aid core biopsy. Three studies with 158 patients assessed the role of OCT in vivo for various RCC types (clear-cell, papillary and chromophobe) as well as oncocytomas [59–61]. All used a commercially available OCT system (Optis™ Integrated System) with two studies assessing diagnostic accuracy [59, 61]. Sensitivity and specificity were reported at 86–91% and 56–75%, with PPV and NPV in the range of 91–97% and 37–56%. These results were inferior compared to standard biopsy; however, two studies identified a higher diagnostic yield of 99% with a decrease of non-diagnostic biopsies by 20% [59, 60]. No evaluation of cost or cost effectiveness was undertaken with no persistent risk of bias assessment concerns identified.

### Penile cancer

#### Optical coherence tomography

A single study assessed the applicability of OCT in penile lesions prior to punch biopsy [62]. This study included 18 patients with a mix of penile intraepithelial lesions (PIN), CIS and squamous cell carcinoma (SCC). This feasibility study, assessed the use of OCT on visible lesions, demonstrating significant differences in terms of epidermal thickness and attenuation coefficient between benign and pre-malignant/malignant lesions. However, no data on diagnostic accuracy or cost were discussed with no risk of bias concerns identified.

#### Photodynamic diagnosis

One study assessed the role of PDD using topical 5-aminolevulinic acid and autofluorescence prior to biopsy of penile CIS or SCC [63]. Twelve patients were assessed with a commercially available system demonstrating clearly defined neoplastic and pre-neoplastic lesions on patients; however, no clear diagnostic accuracy or discussion of cost was reported by the study. Patient selection bias was identified with index test bias due to knowledge of results prior to PDD use seen.

## Discussion

This systematic review provides an overview of the current in vivo evidence base for the use of novel optical imaging modalities in the detection and staging of urological neoplasm. The varying diagnostic accuracies and lack of further characterisation of lesions in current urological optical diagnostic modalities has led to the development of more detailed real-time optical imaging methods that aim to aid intraoperative decision making. However, the current evidence base demonstrates that human in vivo research in this area is still in its infancy with low recommendations of utilisation currently remaining based on our findings (Table 3).

**Table 3:** Summary of GRADE of Recommendation for individual outcome measures in each imaging modality

Imaging modality	Cancer type	Improvement in outcome measure	GRADE of recommendation
Optical coherence tomography	Bladder	Diagnostic accuracy	Low (++)
		Staging of disease	Very Low (+)
	Upper tract	Diagnostic accuracy	Very Low (+)
	Renal biopsy	Diagnostic accuracy	None
		Diagnostic yield	Low (+)
	Penile	Diagnostic accuracy	None
Confocal laser endomicroscopy	Bladder	Diagnostic accuracy	Very low (+)
		Grading	Very low (+)
	Upper tract	Diagnostic accuracy	None
		Grading	None
Autofluorescence	Bladder	Diagnostic accuracy	Very low (+)
Spectroscopies	Bladder	Diagnostic accuracy	Very low (+)
Endocystoscopy	Bladder	Diagnostic accuracy	Very low (+)
Photodynamic diagnosis	Upper tract	Diagnostic accuracy	Low (++)
	Penile	Diagnostic accuracy	None
Narrow band imaging	Upper tract	Diagnostic accuracy	Very low (+)

The largest research interest has been within the context of non-muscle invasive bladder cancer, with OCT the most investigated modality. However, whilst identifying good sensitivities between benign and malignant disease, the widespread use of OCT is limited by several factors. Small study sizes combined with varying systems utilised limit the applicability of these results. Furthermore, there is limited data to support its predominant potential within staging of disease. To increase the clinical applicability of OCT within bladder cancer, further investigation is now required to address this, to demonstrate if it can be used as an intraoperative adjunct which can not only improve diagnosis but also guide treatment. Additionally, a current limitation of its use includes its microscopic field of view, which requires an initial identification of a suspicious area for the placement of the probe and further assessment. Few articles address this limitation to improve its applicability within clinical practice. Further investigation via combination with other adjuncts such as blue light cystoscopy may improve this and thereby improve overall diagnostic accuracy. There is also a need to assess if it is a cost effective modality, ensuring widespread diffusion was feasible. Therefore, it is clear at present that whilst promising data are present, further work is required to not only demonstrate its effectiveness for overall and staging accuracy, but also on how to best utilise it within bladder cancer.

CLE for bladder cancer has generated interest due to its ability to assess tissue at a cellular level intraoperatively, thereby having potential for intraoperative grading and improving diagnostic accuracy. However, at present, there is little objective data to demonstrate this with predominant feasibility of use demonstrated. Once again, evidence is required to demonstrate its predominant clinical applicability within grading of disease which would improve its clinical utility. Additionally, as with OCT, CLE also requires identification of a suspicious lesion prior to utilisation of a probe for assessment. Therefore, its combination with other modalities should be conducted to assess if this improves its diagnostic accuracy, and, therefore, clinical utility. CLE does, however, benefits from the availability of widespread commercial systems available which improves its potential for diffusion if improvements in diagnostic accuracy are proven. At present though its use is still limited, not yet demonstrating to be a useful tool in widespread clinical practice.

The applicability for other imaging methods such as autofluorescence and spectroscopic modalities lie within their ability to offer more clearly defined differentiations between benign and malignant lesions and are, therefore, focused on increasing diagnostic accuracy. However, at present, data demonstrate largely feasibility of these modalities with limited evidence to demonstrate improvements in diagnostic accuracy. This requires assessment against established modalities such as blue light cystoscopy, to establish if they can provide a widespread clinical utility within bladder cancer. Additionally, at present, few commercially available systems are present which would be required if these modalities were to be widely disseminated.

Upper tract malignancy diagnosis provides a challenge with known limitations of endoscopic techniques and inaccurate or non-diagnostic biopsies [64]. There is, therefore, interest in identifying imaging methods that can improve overall diagnostic accuracy. PDD offers a modality which could be widely diffused as established equipment and expertise is already available. It has additionally produced the most consistent evidence within upper tract disease for improved diagnostic accuracy when compared to white light ureteroscopy alone. However, it is clear further data are required with small sample sizes currently present. Further investigation via larger, multi-institutional trials is certainly needed. Additionally, with the difficulties encountered in upper tract diagnosis, it is unlikely PDD will demonstrate a complete solution, and, therefore, its use in conjunction with other modalities such as conventional imaging modalities and cytology assessment is required. This would demonstrate a tool which can be incorporated into current clinical practices and is likely to demonstrate better diagnostic accuracies as opposed to stand-alone use. Other modalities within upper tract disease such as OCT, CLE and NBI presently demonstrate predominant feasibility data. They, therefore, require further diagnostic accuracy assessment, both for overall and for staging/grading of disease prior to further assessment of clinical utility within upper tract disease.

There has been less use of different imaging modalities for other urological malignancies. Three studies assessed the role of OCT for renal biopsies with no additional benefit for diagnostic accuracy with possible benefits for diagnostic yield. However, with commercially available systems widely available and in vivo research in renal biopsy arising only in the last few years, this may change to demonstrate clinical utility by reducing the burden of repeat biopsies. Research within penile cancer at present is only around two isolated studies assessing the role of OCT and PDD, with no information surrounding diagnostic accuracy. It is clear that further initially small study data are required to assess if there is a potential for improving overall diagnostic accuracy within penile cancer prior to utilisation of resources for larger scale trials.

Whilst we present the current evidence base for in vivo human research, there are currently other imaging modalities in development at an earlier stage of assessment which may demonstrate an important role in years to come. Numerous studies have assessed the use of existing modalities such as OCT for use in prostate cancer detection; however, these are at present limited to ex vivo studies [65–68]. Furthermore, new imaging systems such as the Image 1S are currently undergoing validation for non-muscle invasive bladder cancer [69, 70]. Finally, novel imaging methods, including optical molecular imaging such as targeted antibodies for CD47 or pH low insertion peptides (pHLIPs), are being developed and assessed in bladder cancer [71–74]. This means that novel imaging methods in urological malignancy provides an extremely dynamic and developing field which may change diagnostic practices in the future.

The present review offers a comprehensive analysis of current in vivo human studies for novel imaging modalities in urology. Whilst the results of this review have some implications for clinicians in demonstrating a current paucity in data for modalities, they offer more applicability to researchers, highlighting areas of future research in a potentially practice changing field. However, as with any study, this review does have weaknesses. Firstly, despite the comprehensive search strategy, pertinent articles may have been missed which could have impacted the recommendations made. Additionally, studies identified in this narrative review are small, offer a low level of evidence and possess significant heterogeneity in their results. This prevented any meaningful pooling of results via a meta-analysis, preventing statistical estimates of overall diagnostic accuracies for each modality.

## Conclusions

Due to current limitations in diagnosis of urological malignancies, numerous additional optical imaging modalities have been developed and assessed for the detection of neoplastic disease and to provide increased real-time information to guide intraoperative decisions. OCT for bladder cancer and PDD for upper tract malignancy demonstrate the largest potential. However, at present, both still lack the evidence base required for translation into routine clinical practice. Further large and well-designed trials are required for these modalities to assess not only their overall and staging diagnostic accuracies, but also how to best utilise them. Other modalities such as CLE and autofluorescence for bladder cancer and NBI for upper tract disease also demonstrate potential but are at an earlier stage of their investigation. With ongoing research into these and other novel imaging modalities, this promises to be an exciting and dynamic field within urological diagnostics which can potentially improve intra-operative decision making.

## Appendix A: Complete search strategy and results

Optical imaging AND (Cancer OR Carcinoma OR carcinoma in situ OR carcinoma-in-situ OR Neoplasm OR Neoplastic OR Oncology OR Oncological) AND (urology OR urological OR urinary OR bladder OR renal OR kidney OR ureter OR ureteric OR upper urinary tract)

- Pubmed—563
- Embase—270
- Cochrane Library—43
- Total 876

Diagnostic imaging AND (real time OR real-time) AND (in vivo OR in vivo) AND (urology OR urological OR urinary OR bladder OR renal OR kidney OR ureter OR ureteric OR upper urinary tract)

- Pubmed—254
- Embase—25
- Cochrane Library 25
- Total 304

Optical coherence tomography AND (urology OR urological OR urinary OR bladder OR renal OR kidney OR ureter OR ureteric OR ureteric upper urinary tract)

- Pubmed—478
- Embase—990
- Cochrane Library 22
- Total 1490

Confocal Laser Endomicroscopy AND (urology OR urological OR urinary OR bladder OR renal OR kidney OR ureter OR ureteric OR upper urinary tract)

- Pubmed—48
- Embase—114
- Cochrane Library 1
- Total 163

(Near infrared spectroscopy OR near infrared autofluorescence spectroscopy) AND (urology OR urological OR urinary OR bladder OR renal OR kidney OR ureter OR ureteric OR upper urinary tract)

- Pubmed—396
- Embase—596
- Cochrane Library 0
- Total 992

Raman spectroscopy AND (urology OR urological OR urinary OR bladder OR renal OR kidney OR ureter OR ureteric OR upper urinary tract)

- Pubmed—319
- Embase—227
- Cochrane Library 2
- Total 541

High magnification cystoscopy AND (urology OR urological OR urinary OR bladder OR renal OR kidney OR ureter OR ureteric OR upper urinary tract)

- Pubmed—4
- Embase—2
- Cochrane Library 1
- Total 7

Autofluorescence AND (urology OR urological OR urinary OR bladder OR renal OR kidney OR ureter OR ureteric OR upper urinary tract)

- Pubmed—327
- Embase—567
- Cochrane Library 6
- Total 900

Diffuse reflectance spectroscopy AND (urology OR urological OR urinary OR bladder OR renal OR kidney OR ureter OR ureteric OR upper urinary tract)

- Pubmed—17
- Embase—14
- Cochrane Library 1
- Total 32

(Narrow band imaging OR NBI) AND (ureter OR upper urinary tract OR ureteric)

- Pubmed—16
- Embase—36
- Cochrane Library 11
- Total 63

(Photodynamic diagnosis OR PDD) AND (ureter OR upper urinary tract OR ureteric)

- Pubmed—22
- Embase—43
- Cochrane Library—10
- Total 75

Diffuse reflectance spectroscopy AND (urology OR urological OR urinary OR bladder OR renal OR kidney OR ureter OR ureteric OR upper urinary tract)

- Pubmed—17
- Embase—14
- Cochrane Library 1
- Total 32

### Total records screened from databases—5475.

#### Other sources

Ongoing trials identified—5 and contacted, nil returned data.

- JPRN-UMIN000021067
- JPRN-UMIN000017714
- NCT02841904
- NCT03013894
- NCT03013920

Studies identified from reference review of identified articles and reviews—11.
